# Supplementary material for: Modeling glioblastoma heterogeneity as a dynamic network of cell states
Source: Mol Syst Biol. 2021 Sep 16;17(9):e10105. doi: 10.15252/msb.202010105 (PMC8444284; doi:10.15252/msb.202010105)
Supplement: Supplementary file 5 — Source Data for Figure 3 [file MSB-17-e10105-s001.zip › Figure3A_sourcedata/GSEA_3065/hallmarks_state6.GseaPreranked.1623416308702/HALLMARK_UV_RESPONSE_DN.html]

Details for gene set HALLMARK\_UV\_RESPONSE\_DN[GSEA]

|  || Dataset | state6 |
| Phenotype | NoPhenotypeAvailable |
| Upregulated in class | na\_neg |
| GeneSet | HALLMARK\_UV\_RESPONSE\_DN |
| Enrichment Score (ES) | -0.34405106 |
| Normalized Enrichment Score (NES) | -1.3809003 |
| Nominal p-value | 0.03900156 |
| FDR q-value | 0.08495783 |
| FWER p-Value | 0.736 |
Table: GSEA Results Summary

  

Fig 1: Enrichment plot: HALLMARK\_UV\_RESPONSE\_DN      
 Profile of the Running ES Score & Positions of GeneSet Members on the Rank Ordered List

  

| PROBE | GENE SYMBOL | GENE\_TITLE | RANK IN GENE LIST | RANK METRIC SCORE | RUNNING ES | CORE ENRICHMENT || 1 | CITED2 |  |  | 360 | 0.142 | -0.0207 | No |
| 2 | NR3C1 |  |  | 377 | 0.139 | -0.0021 | No |
| 3 | AKT3 |  |  | 616 | 0.112 | -0.0131 | No |
| 4 | APBB2 |  |  | 693 | 0.106 | -0.0062 | No |
| 5 | FYN |  |  | 817 | 0.097 | -0.0063 | No |
| 6 | ATRX |  |  | 861 | 0.094 | 0.0025 | No |
| 7 | DUSP1 |  |  | 924 | 0.090 | 0.0085 | No |
| 8 | ICA1 |  |  | 1102 | 0.080 | -0.0001 | No |
| 9 | NR1D2 |  |  | 1123 | 0.080 | 0.0092 | No |
| 10 | CELF2 |  |  | 1137 | 0.079 | 0.0192 | No |
| 11 | FZD2 |  |  | 1178 | 0.076 | 0.0258 | No |
| 12 | RND3 |  |  | 1213 | 0.075 | 0.0328 | No |
| 13 | PIK3R3 |  |  | 1321 | 0.070 | 0.0308 | No |
| 14 | PRKAR2B |  |  | 1482 | 0.065 | 0.0219 | No |
| 15 | MTA1 |  |  | 1542 | 0.062 | 0.0242 | No |
| 16 | KALRN |  |  | 1543 | 0.062 | 0.0333 | No |
| 17 | TJP1 |  |  | 1659 | 0.058 | 0.0286 | No |
| 18 | PTEN |  |  | 1739 | 0.055 | 0.0276 | No |
| 19 | MAP2K5 |  |  | 1819 | 0.053 | 0.0262 | No |
| 20 | AGGF1 |  |  | 1827 | 0.053 | 0.0331 | No |
| 21 | BHLHE40 |  |  | 1880 | 0.051 | 0.0346 | No |
| 22 | SPOP |  |  | 1917 | 0.050 | 0.0378 | No |
| 23 | MYC |  |  | 2081 | 0.045 | 0.0257 | No |
| 24 | BMPR1A |  |  | 2099 | 0.045 | 0.0303 | No |
| 25 | ZMIZ1 |  |  | 2143 | 0.043 | 0.0317 | No |
| 26 | RXRA |  |  | 2249 | 0.040 | 0.0255 | No |
| 27 | SIPA1L1 |  |  | 2251 | 0.040 | 0.0312 | No |
| 28 | ANXA4 |  |  | 2272 | 0.039 | 0.0346 | No |
| 29 | CDC42BPA |  |  | 2279 | 0.039 | 0.0397 | No |
| 30 | ACVR2A |  |  | 2368 | 0.037 | 0.0349 | No |
| 31 | ID1 |  |  | 2451 | 0.035 | 0.0306 | No |
| 32 | MGLL |  |  | 2542 | 0.032 | 0.0250 | No |
| 33 | PHF3 |  |  | 2709 | 0.029 | 0.0100 | No |
| 34 | NFIB |  |  | 2762 | 0.028 | 0.0081 | No |
| 35 | MAP1B |  |  | 2893 | 0.024 | -0.0033 | No |
| 36 | PLPP3 |  |  | 2894 | 0.024 | 0.0002 | No |
| 37 | EFEMP1 |  |  | 3173 | 0.018 | -0.0291 | No |
| 38 | VLDLR |  |  | 3225 | 0.017 | -0.0325 | No |
| 39 | YTHDC1 |  |  | 3286 | 0.016 | -0.0371 | No |
| 40 | FHL2 |  |  | 3383 | 0.013 | -0.0462 | No |
| 41 | ARHGEF9 |  |  | 3407 | 0.013 | -0.0469 | No |
| 42 | SCHIP1 |  |  | 3493 | 0.011 | -0.0551 | No |
| 43 | DMAC2L |  |  | 3714 | 0.006 | -0.0794 | No |
| 44 | SERPINE1 |  |  | 3811 | 0.004 | -0.0898 | No |
| 45 | MRPS31 |  |  | 3861 | 0.003 | -0.0950 | No |
| 46 | IGF1R |  |  | 3930 | 0.002 | -0.1025 | No |
| 47 | RUNX1 |  |  | 3950 | 0.001 | -0.1045 | No |
| 48 | MIOS |  |  | 3965 | 0.001 | -0.1059 | No |
| 49 | SMAD3 |  |  | 4246 | -0.004 | -0.1376 | No |
| 50 | DDAH1 |  |  | 4446 | -0.008 | -0.1593 | No |
| 51 | CDKN1B |  |  | 4475 | -0.008 | -0.1613 | No |
| 52 | CAP2 |  |  | 4567 | -0.010 | -0.1703 | No |
| 53 | NOTCH2 |  |  | 4874 | -0.016 | -0.2032 | No |
| 54 | NFKB1 |  |  | 4949 | -0.017 | -0.2092 | No |
| 55 | VAV2 |  |  | 5183 | -0.022 | -0.2328 | No |
| 56 | RASA2 |  |  | 5246 | -0.023 | -0.2365 | No |
| 57 | PRDM2 |  |  | 5270 | -0.024 | -0.2357 | No |
| 58 | AMPH |  |  | 5496 | -0.028 | -0.2574 | No |
| 59 | IRS1 |  |  | 5541 | -0.029 | -0.2582 | No |
| 60 | ATRN |  |  | 5791 | -0.034 | -0.2819 | No |
| 61 | MAPK14 |  |  | 5821 | -0.035 | -0.2801 | No |
| 62 | KCNMA1 |  |  | 5915 | -0.036 | -0.2855 | No |
| 63 | BDNF |  |  | 6042 | -0.039 | -0.2943 | No |
| 64 | SRI |  |  | 6182 | -0.042 | -0.3041 | No |
| 65 | RBPMS |  |  | 6189 | -0.042 | -0.2986 | No |
| 66 | PDLIM5 |  |  | 6585 | -0.052 | -0.3364 | Yes |
| 67 | SDC2 |  |  | 6588 | -0.053 | -0.3289 | Yes |
| 68 | MET |  |  | 6611 | -0.053 | -0.3237 | Yes |
| 69 | TGFBR2 |  |  | 6727 | -0.057 | -0.3287 | Yes |
| 70 | MAGI2 |  |  | 6760 | -0.058 | -0.3239 | Yes |
| 71 | NIPBL |  |  | 6763 | -0.058 | -0.3157 | Yes |
| 72 | TENT4A |  |  | 6790 | -0.058 | -0.3101 | Yes |
| 73 | ADD3 |  |  | 6818 | -0.059 | -0.3046 | Yes |
| 74 | GJA1 |  |  | 6876 | -0.061 | -0.3022 | Yes |
| 75 | IGFBP5 |  |  | 7011 | -0.065 | -0.3081 | Yes |
| 76 | SFMBT1 |  |  | 7050 | -0.066 | -0.3028 | Yes |
| 77 | SCAF8 |  |  | 7095 | -0.068 | -0.2980 | Yes |
| 78 | SLC22A18 |  |  | 7123 | -0.069 | -0.2910 | Yes |
| 79 | ATP2C1 |  |  | 7132 | -0.069 | -0.2819 | Yes |
| 80 | SYNE1 |  |  | 7282 | -0.074 | -0.2883 | Yes |
| 81 | BCKDHB |  |  | 7286 | -0.074 | -0.2778 | Yes |
| 82 | TFPI |  |  | 7359 | -0.076 | -0.2749 | Yes |
| 83 | CDK13 |  |  | 7396 | -0.078 | -0.2677 | Yes |
| 84 | ERBB2 |  |  | 7405 | -0.078 | -0.2573 | Yes |
| 85 | ADORA2B |  |  | 7469 | -0.080 | -0.2528 | Yes |
| 86 | SLC7A1 |  |  | 7535 | -0.083 | -0.2481 | Yes |
| 87 | HAS2 |  |  | 7560 | -0.084 | -0.2386 | Yes |
| 88 | LDLR |  |  | 7648 | -0.088 | -0.2357 | Yes |
| 89 | INSIG1 |  |  | 7721 | -0.092 | -0.2305 | Yes |
| 90 | DYRK1A |  |  | 7781 | -0.094 | -0.2235 | Yes |
| 91 | ATP2B4 |  |  | 7858 | -0.099 | -0.2178 | Yes |
| 92 | MMP16 |  |  | 7971 | -0.105 | -0.2153 | Yes |
| 93 | DLG1 |  |  | 8081 | -0.112 | -0.2114 | Yes |
| 94 | CAV1 |  |  | 8156 | -0.117 | -0.2028 | Yes |
| 95 | ATP2B1 |  |  | 8436 | -0.150 | -0.2129 | Yes |
| 96 | ATXN1 |  |  | 8456 | -0.153 | -0.1927 | Yes |
| 97 | PRKCA |  |  | 8485 | -0.158 | -0.1729 | Yes |
| 98 | COL5A2 |  |  | 8514 | -0.165 | -0.1520 | Yes |
| 99 | COL11A1 |  |  | 8518 | -0.165 | -0.1282 | Yes |
| 100 | PMP22 |  |  | 8560 | -0.175 | -0.1073 | Yes |
| 101 | LAMC1 |  |  | 8618 | -0.199 | -0.0848 | Yes |
| 102 | CACNA1A |  |  | 8642 | -0.211 | -0.0567 | Yes |
| 103 | SYNJ2 |  |  | 8680 | -0.228 | -0.0276 | Yes |
| 104 | ANXA2 |  |  | 8741 | -0.279 | 0.0063 | Yes |
Table: GSEA details [plain text format]

  

Fig 2: HALLMARK\_UV\_RESPONSE\_DN: Random ES distribution      
 Gene set null distribution of ES for **HALLMARK\_UV\_RESPONSE\_DN**
